# Lipid Discovery by Combinatorial Screening and Untargeted LC-MS/MS

**DOI:** 10.1038/srep27920

**Published:** 2016-06-17

**Authors:** Mesut Bilgin, Petra Born, Filomena Fezza, Michael Heimes, Nicolina Mastrangelo, Nicolai Wagner, Carsten Schultz, Mauro Maccarrone, Suzanne Eaton, André Nadler, Matthias Wilm, Andrej Shevchenko

**Affiliations:** 1 Max Planck Institute for Cell Biology and Genetics, Pfotenhauerstraβe 108, 01307 Dresden, Germany; 2 Department of Experimental Medicine and Surgery, Tor Vergata University of Rome, via Montpellier 1, 00133, Rome, Italy; 3 European Center for Brain Research/Fondazione Santa Lucia, via del Fosso di Fiorano 65, 00143 Rome, Italy; 4 European Molecular Biology Laboratory (EMBL), Meyerhofstrasse 1, 69117 Heidelberg, Germany; 5 Department of Medicine, Campus Bio-Medico University of Rome, Via Alvaro del Portillo 21, 00128 Rome; 6 Conway Institute of Biomolecular and Biomedical Research, University College Dublin, 4 Dublin, Ireland

## Abstract

We present a method for the systematic identification of picogram quantities of new lipids in total extracts of tissues and fluids. It relies on the modularity of lipid structures and applies all-ions fragmentation LC-MS/MS and Arcadiate software to recognize individual modules originating from the same lipid precursor of known or assumed structure. In this way it alleviates the need to recognize and fragment very low abundant precursors of novel molecules in complex lipid extracts. In a single analysis of rat kidney extract the method identified 58 known and discovered 74 novel endogenous endocannabinoids and endocannabinoid-related molecules, including a novel class of *N*-acylaspartates that inhibit Hedgehog signaling while having no impact on endocannabinoid receptors.

Cells produce a palette of lipid bioregulators, *e.g.* steroid hormones, eicosanoids or endocannabinoids, to mention only a few. Known molecules may be identified by probing MS/MS spectra against a reference spectra database^1^ or by their direct interpretation relying upon common fragmentation mechanisms and lipid-class specific compositional constraints^2^. In principle, these approaches could be extended to the identification of putative lipid structures by relaxing spectra matching requirements^3^,^4^. However, in crude extracts the abundance of new molecules is often too low compared to other lipids. Therefore their precursor ions are not selected for fragmentation in data-dependent LC-MS/MS experiments and in shotgun experiments they render non-interpretable MS/MS spectra dominated by product ions of co-fragmented lipids and chemical noise. This constitutes a major bottleneck in a systematic discovery of novel lipids.

Despite their compositional diversity, lipids are composed from a large, yet finite compendium of common structural modules such as fatty acids, amino acids, glycerol, ethanolamine, carbohydrates, *etc.*, which associate in numerous combinations^5^. Consistent with the modular organization, MS/MS fragmentation of lipid precursors produces signature ions specific for each structural module and their grouping defines the lipid classes and individual species^2^,^6^. To recognize and group the signature ions it might not be necessary to mass-select each precursor individually. Instead, all precursors co-eluted during LC-MS/MS experiment may be fragmented at once and all fragments simultaneously detected in a highly convoluted spectrum – this approach was termed as MS^E^ and also as all-ions fragmentation LC-MS/MS (AIF LC-MS/MS)^7^,^8^,^9^,^10^. Fragment ions could be associated with corresponding precursors by retroactive *in silico* alignment of their peaks in extracted ion chromatograms (XIC). We implemented AIF LC-MS/MS in an unbiased screening approach and showed that it could systematically identify novel lipid molecules and lipid classes at the low picogram level.

## Results

**Figure 1:**
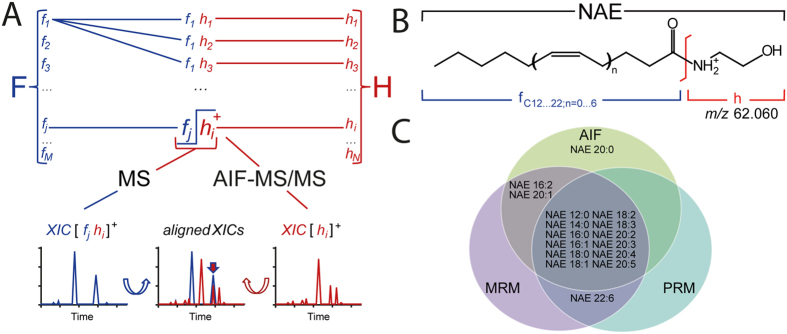
Design and validation of the lipid discovery workflow. (**A**) AIF LC-MS/MS screen for molecules consisting of a polar head group H and fatty acid moiety F. A putative compound *f*_*j*_*h*_*i*_ is identified if XIC peaks of its precursor ion [*f*_*j*_*h*_*i*_]^+^ (in blue) and fragment [*h*_*i*_]^+^ spectra (in red) align (two-colour arrow). (**B**) Molecular ion and the head group fragment of *N*-acylethanolamines (NAE). (**C**) Venn diagram of endogenous NAE independently identified by all-ions fragmentation (AIF) and parallel reaction monitoring (PRM) on a Q Exactive and by multiple reaction monitoring (MRM) on a triple quadrupole mass spectrometer.

We reasoned that novel lipid structures may arise from yet unknown combinations of already known modules and both known and novel combinations may be recognized in the same AIF LC-MS/MS analysis (Fig. 1). For presentation clarity, let us assume that anticipated lipid molecules may consist of two structural modules: a polar head group (H) and a fatty acid (F). To screen for new molecules, we will first design plausible structures by combining all fatty acid moieties *f*_*j*_ with all head groups *h*_*i*_ from the H and F lists, respectively. For each compound *f*_*j*_*h*_*i*_ we compute *m/z* of its precursor ion [*f*_*j*_*h*_*i*_]^+^ and characteristic polar head group fragment [*h*_*i*_]^+^ which would be most likely detectable in tandem mass spectra. AIF LC-MS/MS of a total extract detects all ionisable precursors (in MS spectra) and all fragments (in AIF MS/MS spectra). Chromatographic peaks of anticipated precursors (*XIC* [*f*_*j*_*h*_*i*_]^+^) and fragments (*XIC* [*h*_*i*_]^+^) are then aligned by the in-house developed Arcadiate software. They should neatly overlap if [*h*_*i*_]^+^ is produced from [*f*_*j*_*h*_*i*_]^+^ (see *aligned XIC* trace in Fig. 1A), while no or partial match indicates that this pair of ions is unrelated. The same scheme also applies for more complex fragmentation patterns since peaks alignment specificity increases if XICs of several fragments are required to match. A dataset produced by a single AIF LC-MS/MS experiment suffices for *in silico* screening for an unrestricted number of combinations of modules. If the structure of candidate molecules remains ambiguous, AIF LC-MS/MS should be followed by the targeted acquisition of full high resolution MS/MS spectra of their precursors, chemical derivatization^11^ and, ultimately, by chemical synthesis.

We applied AIF LC-MS/MS to profile endogenous endocannabinoid-related compounds (ERC) in a total extract of rat kidney (Fig. 1A). ERC consist of two structural modules: a fatty acid or fatty alcohol linked to a polar head group (which determines its class), such as ethanolamine, glycerol, dopamine or amino acids (such as Ser or Gly). *Bona fide* endocannabinoids are physiological ligands of type 1 and type 2 cannabinoid receptors (CB_1_R and CB_2_R) and implicated in neurological, metabolic and cardiovascular diseases (reviewed in)^12^. ERC are widespread across phyla^13^,^14^ and have a broad spectrum of activities^15^,^16^. They are present in fluids and tissues at the nM or picogram per mg range^17^,^18^, respectively, and therefore common LC-MS/MS analyses only target a few key molecules. ERC are structurally diverse, however no method afforded a comprehensive overview of their composition: although in total more than 80 ERC have been identified this list is certainly not exhaustive^16^,^19^,^20^.

We first benchmarked our method by comparing the number of *N*-acylethanolamines (NAE) identified by AIF and, independently, by the two targeted LC-MS/MS methods^17^,^21^ – multiple reaction monitoring (MRM) on a triple quadrupole mass spectrometer and by parallel reaction monitoring (PRM) on a hybrid high resolution tandem mass spectrometer Q Exactive (Fig. 1B). Both MRM and PRM rely upon an inclusion list of targeted precursors compiled prior the analysis. Contrary, AIF profiling is unbiased since a full dataset of MS and MS/MS spectra is acquired first and then XIC traces for any desired combination of precursor and fragment masses are retrieved and aligned. For PRM and MRM analyses we compiled a list of *m/z* of 19 candidate NAE molecules that included species with common C16-C20 fatty acids with 0 to 6 double bonds; we also added two shorter saturated C12 and C14 fatty acids and three polyunsaturated C22 fatty acids with 4 to 6 double bonds (Supplementary Table S1). This list comprised all currently known NAE species and a few plausible candidates comprising common fatty acid moieties. Considering even larger number of candidate molecules could compromise the sensitivity of both reference methods, while having no impact on the sensitivity of AIF. In all three experiments NAE were identified by detecting the ethanolamine head group fragment (*m/z* 62.060) produced by MS/MS of 19 expected precursor ions (Supplementary Table S1). We note that, although MRM and PRM relied on the same mass transitions, they were performed independently on different instruments and were complementary. MRM was expected to have higher sensitivity, yet lower specificity in selecting precursor and fragment *m/z*, while PRM was performed at much higher mass resolution and therefore should be detecting precursors and fragments with higher specificity albeit lower sensitivity.

In a rat kidney extract AIF LC-MS/MS identified 15 NAE molecules, including 12 out of 13 molecules found by both MRM and PRM (Fig. 1C). In total, it recognized the same number of NAE molecules (15) as the method of MRM. It failed to detect NAE 22:6 because of background interference; this, however, was unrelated to the high degree of unsaturation of its fatty acid moiety. Hence the identification of endogenous NAE by the untargeted AIF and both targeted (MRM, PRM) LC-MS/MS methods was consistent.

**Figure 2:**
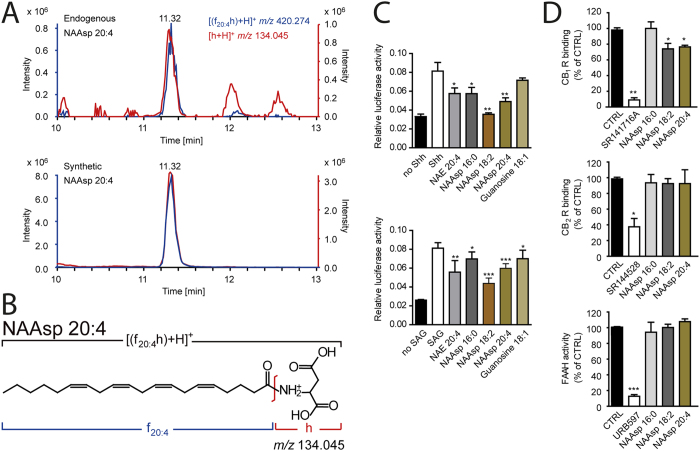
Identification and biological activity of N-acylaspartates. (**A**) Alignment of XIC peaks of the [M+H]^+^ precursor and head group fragment (upper panel) identified NAAsp 20:4, consistently with the structure of its molecular ion (**B**) and independent analysis of the synthesised molecule (lower panel). (**C**) Shh-LIGHT2 cell reporter assay showing that NAAsp inhibit Hedgehog (Hh) signalling. Hh activation after stimulation with Smoothened agonist (SAG) or non-sterol-modified Shh was reduced by 15 μM of NAAsp 16:0; NAAsp 18:2; NAAsp 20:4; NAE 20:4 (positive control) and *O*-acylguanosine 18:1 (negative control) (*n* = 3). (**D**) NAAsp (10 μM) did not interact with CB_2_ and very weakly with CB_1_ receptors; they also did not inhibit FAAH. Ctrl stands for negative control; SR141716A (1 μM); SR144528 (1 μM) and URB597 (0.1 μM) are synthetic blockers serving as positive controls. (*n* = 4; ^*^p < 0.05; ^**^p < 0.001 and ^***^p < 0.0005 *vs* control).

We further hypothesized that ERC might feature a broader selection of polar head groups and fatty acids/alcohols, yet sharing the same modular organization. In principle, amino- and hydroxyl groups of any endogenous small molecule are plausible targets for *N*-acylation, *O*-esterification or -etherification, respectively. We therefore selected 52 polar head groups including amino acids, nucleobases and biogenic amines, each of which was conjugated *in silico* to 39 fatty acid/fatty alcohol moieties (Supplementary Dataset 1), altogether representing over 2000 putative molecules. A single AIF LC-MS/MS run of rat kidney extract recognized 132 ERC of 18 individual classes, out of which 74 molecules were novel (Supplementary Table S2). Furthermore, we identified 3 molecules of a novel class of ERC: *N*-acylaspartates (NAAsp) having 16:0, 18:2 and 20:4 fatty acid moieties attached to the amino group of aspartic acid (Fig. 2). Considering an extended list of uncommon fatty acids (*e.g.* having odd number of carbon atoms or a hydroxyl group) produced no further hits.

To further validate the identification these three NAAsp were synthesized. We found that their retention times and ratio of abundances of precursor and fragment peaks were the same as of endogenous molecules (Fig. 2A,B).

How abundant were NAAsp molecules compared to other endogenous ERC? We quantified NAAsp and four major ERC classes (1-acylglycerols, 2-acylglycerols, NAE and *N*-acylglycines)^22^ in the same extract. NAAsp was 2-fold less abundant than the least abundant endocannabinoid class of NAE (including anandamide) and 100-fold less abundant than 1-acylglycerols (Supplementary Table S3), which underscores the superior sensitivity of AIF LC-MS/MS.

We recently reported that anandamide (NAE 20:4) inhibits Hedgehog (Hh) signalling by binding the 7-pass transmembrane protein Smoothened^15^. To ask whether NAAsp may also interfere with Hh, we compared NAE 20:4 with NAAsp for their capacity to block both Sonic Hedgehog (Shh) and SAG-induced Smoothened activation (Fig. 2C). NAAsp 20:4 and NAAsp 18:2 were as active as anandamide in both assays. In contrast to anandamide, NAAsp do not bind CB_2_R, only weakly bind CB_1_R, and are resistant to major anandamide-degrading enzyme FAAH^23^ (Fig. 2D). This makes NAAsp promising candidates for pharmacological targeting of the Hedgehog signalling pathway.

## Discussion

We developed a method to rapidly screen for novel endogenous lipid molecules in complex biological extracts featuring notably improved sensitivity compared to “gold standard” targeted mass spectrometric analyses by MRM and PRM. Our method is unbiased and extendable to any known or plausible combination of lipid structural modules. AIF LC-MS/MS provides a rich dataset that comprises all fragment ions produced from all ionisable precursors, including fragments originating *via* novel or unexpected fragmentation pathways and intermolecular rearrangements. Conceivably, it might be possible to implement a multi-layer datamining strategy that considers alternative combinations of fragments linked by *boolean* logic operations^24^.

We underscore that the major bottleneck of the identification of picogram amount of lipids is not the acquisition of informative MS/MS spectra or database searches, but rather in recognizing and selecting the appropriate precursor ion for the subsequent fragmentation. We demonstrated that it is circumvented by untargeted fragmentation of all ionised precursors and the complexity of highly convoluted all-fragments tandem mass spectra is compensated by high mass resolution, spectra acquisition rate and dynamic range of the Orbitrap mass analyzer. AIF LC-MS/MS outperformed targeted analytical approaches and identified all known and also a new class of ECR at the concentration levels 2-fold below the least abundant genuine EC.

We also speculate that we might have acquired traces of many more novel compounds, whose identification would require better datamining that could automatically produce and evaluate a large number of putative structures. Alternatively, it could identify candidate molecules by unbiased alignment of XICs of unspecified fragments. Altogether, it paves the way for a comprehensive and systematic characterization of endogenous lipid bioregulators in tissues and fluids and understanding their role in health and disease^25^.

## Materials and Methods

### Chemicals and standards

LC grade solvents and common chemicals (ACS grade) were purchased from Sigma-Aldrich (Munich, Germany) and Fisher Scientific (Schwerte, Germany). Standards of endogenous and deuterium-labeled endocannabinoids, synthetic blockers used in endocannabinoid activity assays (Fig. 2) were purchased from Cayman Chemical Company (Ann Arbor, MI) and Tocris Bioscience (Bristol, UK).

### Extraction and quantification of ERC

By LC-MS/MS on a triple quadrupole mass spectrometer TSQ Vantage (Thermo Fisher Scientific, San Jose CA) was performed as described^22^ with minor modifications; MRM transitions and instrument settings are in Supplementary Table S1. NAAsp were quantified by external calibration using unlabeled synthetic compounds. To compensate matrix effects standards were spiked into a rat tissue extract in which NAAsp were undetectable. Comparative profiling of NAE by the method of PRM on a hybrid quadrupole – Orbitrap tandem mass spectrometer Q Exactive (Thermo Fisher Scientific) was performed as described^22^ using inclusion list of precursor *m/z* (Supplementary Table S1). Rat tissues were obtained from licensed Biomedical Services Facility (MPI of Molecular Cell Biology and Genetics, Dresden). Rat tissues were provided by the animal facility of the Max Planck Institute of Molecular Cell Biology and Genetics, Dresden, Germany in compliance with German animal welfare legislation and in strict pathogen-free conditions. Animal handling protocols were approved by the Institutional Animal Welfare Officer (Tierschutzbeauftragter), and necessary licenses were obtained from the regional Ethical Commission for Animal Experimentation of Dresden, Germany (Tierversuchskommission Landesdirektion Sachsen).

### Software for AIF LC-MS/MS discovery screening

AIF LC-MS/MS spectra were processed by Arcadiate 2.0 software, a tool for visualization and analysis of LC-MS/MS data. Arcadiate is available for download at: https://itunes.apple.com/de/app/arcadiate/id585258468?mt=12&uo=4

Arcadiate operates under a Macintosh OS v.10.8 or higher and requires a desktop PC with minimum of 4-core processor and 4 GB memory. LC-MS/MS (.raw) files should be converted to mzML or mzXML format prior processing. In the benchmarking experiment, importing and generating a chromatographic map from 520 MB mzXML file containing 1015 MS and 1016 MS/MS spectra required *ca* two minutes on a desktop PC (16 GB 1600 MHz RAM; 2.0 GHz Quad-Core Intel i7 processor). Importing a .csv library of *ca* 4000 masses of precursor and fragment ions of compound candidates and associating them with the chromatographic map took a few seconds.

### Identification of ERC by AIF LC-MS/MS

The acquisition cycle consisted of FT MS and FT-MS/MS scans in positive ion mode. FT MS spectra were acquired in the *m/z* range of 225 to 725 at the mass resolution of R_*m/z* 200_ = 140,000; automated gain control (AGC) value of 1 × 10^6^; maximum injection time of 1024 ms. All-ions FT MS/MS were acquired from the same *m/z* range of precursors. Fragments were detected within *m/z* 50 to 725 at R_*m/z* 200_ = 17,500 with AGC value 3 × 10^6^; maximum injection time of 1024 ms; nCE was step increased at 20, 25 and 35%. Spectra in .raw format were converted to mzML and interpreted by Arcadiate software. It aligned XIC peaks of putative precursors and fragments (Supplementary Dataset S1) and sorted the alignments according to their quality (see Supplementary Text for algorithm details). Hits with the alignment score below 50% were discarded.

### Chemical synthesis of NAAsp

NAAsp were synthesized in a two-step procedure. In a first step, the respective fatty acids were coupled to bis-*tert*-butyl protected aspartic acid via EDC/DMAP mediated amide bond formation. The *tert*-butyl groups were subsequently removed by treatment with TFA thus yielding the respective NAAsp. For experimental details, please see the Supplementary Information.

### NAAsp binding to CB_1_R and CB_2_R, and FAAH assay

Binding to CB_1_ and CB_2_ receptors was quantified by displacement of [^3^H]CP55.940 from membrane fractions isolated from mouse brain (as a source of CB_1_R) or mouse spleen (as a source of CB_2_R). FAAH activity was assayed in brain homogenates with 10 μM [ethanolamine-^14^C]anandamide and the release of [ethanolamine-^14^C] in the aqueous phase was measured. Details of experimental protocols are in Supplementary Materials.

### Inhibition of Hedgehog signalling by NAAsp

The effect of NAAsp on Hh signalling pathway was tested in the Shh-LIGHT2 reporter assay as described^15^. The tested compounds were added together with non-sterol-modified Sonic Hedgehog (Shh) or Smoothened agonist (SAG) to LIGHT2 cells. Luciferase activity was measured in cell lysates and Hh pathway activity was estimated as a ratio between Firefly: Renilla luciferase (see Supplementary Materials for details).

## Additional Information

**How to cite this article**: Bilgin, M. *et al*. Lipid Discovery by Combinatorial Screening and Untargeted LC-MS/MS. *Sci. Rep.* **6**, 27920; doi: 10.1038/srep27920 (2016).

## Supplementary Material

Supplementary Information

Supplementary Dataset 1
