# Supplementary material for: Lipid Discovery by Combinatorial Screening and Untargeted LC-MS/MS
Source: Sci Rep. 2016 Jun 17;6:27920. doi: 10.1038/srep27920 (PMC4911551; doi:10.1038/srep27920)
Supplement: Supplementary Information [file srep27920-s1.pdf]

Supplementary Materials

for the manuscript by Mesut Bilgin, Petra Born, Filomena Fezza, Michael Heimes, Nicolina Mastrangelo, Nicolai Wagner, Carsten Schultz, Mauro Maccarrone, Suzanne Eaton, Andre Nadler, Matthias Wilm and Andrej Shevchenko **“Lipid Discovery by Combinatorial Screening and Untargeted LC-MS/MS”**

Table of Contents

1. Supplementary Methods

2. Supplementary Table S1. Identification of *N*-acylethanolamines by multiple reaction monitoring (MRM) and parallel reaction monitoring (PRM) on the TSQ Vantage and Q Exactive mass spectrometers, respectively.

3. Supplementary Table S2. Endocannabinoid-related compounds identified in a rat kidney extract by AIF LC-MS/MS.

4. Supplementary Table S3. Quantification of major classes and individual molecules of endocannabinoid related compounds in the rat kidney extract

Supplementary Methods

Chemicals and standards

Solvents of LC grade were purchased from Sigma-Aldrich (Munich, Germany) and Fisher Scientific (Schwerte, Germany). Endocannabinoid standards were from Cayman Chemical Company (Ann Arbor, MI).

Rat kidney homogenization

To 500µL of 0.1% formic acid in H_2O were added to 50 mg of rat kidney tissue and the sample was twice homogenized at 4°C on TissueLyser II (QIAGEN) with 30 freq/sec for 1.5 min with 30 sec break.

Extraction of endocannabinoid-related compounds

Extraction was performed at 4 °C in a cold room. 750 µl of ethyl acetate/*n*-hexane (9:1 v/v) containing 0.1% formic acid were added to the kidney homogenate and vortexed for 30 sec followed by 10 min centrifugation at 14,000 g. Samples were incubated on dry ice for 10 min and the upper (organic) phase was collected and dried in a vacuum centrifuge. The samples were re-dissolved in 90 µL of water/ acetonitrile/ *iso*-propanol/ formic acid (6:3.6:0.4:0.1, v/v/v/v) mixture, centrifuged for 5 min at 14,000 g, transferred into a new Eppendorf tube, centrifuged for 5 min at 14,000 g and then 90 µL of the solution transferred into a 300 µL glass vial for LC-MS/MS analyses.

Quantification of endogenous endocannabinoids

Fifty µL of internal standard mixture consisting of 60 nM of d4-N-acylethanolamide 16:0; 191.5 nM of d4-N-acylethanolamide 18:2; 146.1 nM of d8-*N*-acylethanolamide 20:4; 400 nM of d8-N-acylglycine 20:4; 50 nM of d8-2-acylglycerol 20:4 and 400 nM of d5-1-acylglycerol 20:4 were added to the homogenate prior extraction. Dried extracts were re-dissolved in 90 µL of water/ acetonitrile/ iso-propanol/ formic acid (6:3.6:0.4:0.1; v/v/v/v) mixture, centrifuged for 5 min at 14 000 g, transferred into an Eppendorf tube, centrifuged for 5 min at 14 000 g and then 90 µL of the solution were transferred into a 300 µL glass vial for LC-MS/MS analysis (see below). Concentrations of endogenous endocannabinoids were estimated by the comparison of abundance of their XIC peaks with the peaks of isotopically labelled standards.

HPLC was performed on an Agilent LC 1100 system (Amstelveen, The Netherlands) We used C4 2 μL trap column (optipak) in-line with 0.5 mm x 150 mm C18 Zorbax analytical column (5 μm particles); the flow rate of 20 μL/min; injection volume of 40 μL. The elution gradient was composed from the solvent A: 0.1% formic acid in water; solvent B: acetonitrile / iso-propanol/formic acid (9:1:0.1 v/v/v). The gradient profile was: 0 min, 40% B; 0–5 min, 40% B; 5–7 min, 40-66.4% B; 7–13 min, 66.4-73% B; 13-15 min, 73-95% B; 15-19 min, 95% B; 19-20 min, 40% B (isocratic); 20-24 min, 40%.

N-acylaspartate endocannbinoid activity assays

Anandamide (AEA) and CP55.940 were purchased from Sigma Chemical Co. (St. Louis, MO, USA), URB597 was obtained from Cayman Chemical (Ann Arbor, MI, USA), SR141716A and SR144528 were purchased from Tocris Bioscience (Bristol, U.K.). [^{3}H]CP55.940 (126 Ci/mmol) was from Perkin-Elmer Life Sciences, Inc. (Boston, MA). [^{14}C-ethanolamine]anandamide (60 Ci/mmol) was purchased from ARC (St. Louis, MO).

a) N-acylaspartate binding to CB_1R nd CB_2R

For cannabinoid receptor studies, tissues were resuspended in 2 mM Tris-EDTA, 320 mM sucrose, 5 mM $MgCl_2$ (pH 7.4), and then they were homogenized in a Potter homogenizer and centrifuged at 4°C sequentially at 800x*g* (10 min), and 10,000x*g* (30 min). The resulting pellet was resuspended in assay buffer (50 mM Tris-HCl, 2 mM Tris-EDTA, 3 mM $MgCl_2$, pH 7.4). The membrane preparation was divided in aliquots, quickly frozen in liquid nitrogen, and stored at -80 °C for no longer than 1 week. The membrane fractions (100 µg per test) were used in rapid filtration assays with the synthetic cannabinoid [^{3}H]CP55.940. In all assays, homogenates were pre-incubated for 15 min with each compound tested. In all experiments, unspecific binding was determined in the presence of an excess (1 µM) of ‘‘cold’’ agonist, and also in the presence of selective antagonists (SR141716A for CB_1 and SR144528 for CB_2) (Pucci et al., 2012).

b) Inhibition of FAAH by N-acylaspartate

Mouse brain homogenates (40 µg per test) were pre-incubated for 15 min at room temperature with each compound tested, then they were incubated with 10 µM [^{14}C-ethanolamine]anandamide for 15 min at 37 °C, in 500 µl of 50 mM Tris–HCl buffer (pH = 9). The reaction was stopped by the addition of 1 mL of

ice-cold methanol/chloroform (2:1, v/v). The mixture was centrifuged at 3000x*g* for 5 min, the upper aqueous layer was put in a vial containing liquid scintillation cocktail (Ultima Gold XR, Perkin Elmer Life Sciences), and radioactivity was quantified in a β-counter. FAAH activity was expressed as pmol [^{14}C-ethanolamine] released/min per mg of protein (Gattinoni et al., 2010).

Inhibitory activity of N-acylasparates against Hh signalling

Shh-LIGHT2 cells (Taipale, 2000), which represent a reporter for Hedgehog (Hh) signalling pathway activity, were maintained in Dulbecco's Modified Eagle medium (DMEM) supplemented with 10% fetal calf serum (FCS), 150 µg/mL Zeocin (Invitrogen), and 400 µg/mL G418 (Invitrogen). Twenty-four hrs prior to assay, cells were plated at a density of 70,000 cells per well in 96-well plates. Cells were then switched to serum-free medium (DMEM + 1% ITS-X) and supplemented with conditioned medium from mock transfected HeLa cells (background), Sonic Hedgehog (Shh) transfected HeLa cells (which release a processed but non-sterol-modified form of Shh), Smoothened agonist (SAG) (Cayman) or DMSO (background). Shh and SAG activities were assayed in the presence or absence of different endocannabinoid classes and species. 20:4 ethanolamide (NAE 20:4) served as a control (Khaliullina, 2015). Luciferase activity was measured in cell lysates after 24 hrs, as instructed by the manufacturer (Dual Glo Luciferase Assay, Promega). The resulting Hedgehog (Hh) pathway activity was determined as the ratio between Firefly : Renilla luciferase.

XIC alignment algorithm employed by the Arcadiate software

Arcadiate employs an original two-step XIC alignment algorithm. First, for every precursor a global correlative chromatogram is produced by computing the average of natural logarithms of intensities of the precursor and its fragments ions. The chromatogram identifies time ranges where precursor and fragments are detected simultaneously. Second, individual time shift values are computed by aligning XIC peaks of the precursor and its fragments within close proximity of the precursor peak. Overall, the alignment quality is assessed by a global detection score expressed in % of fragments with a successful time - based correlation to their precursor. If the time-shift of the optimal overlap between the precursor and a corresponding fragment peak candidate is less than the estimated chromatographic peak width, then the fragment is scored as detected (value 1). If the time shift is larger or the signal intensity of the fragment exceeds the precursor intensity, then it is scored as non-detected (value 0). The overall score termed as

molecular marker detection score is computed by averaging the scores of all fragments and expressed in %. A molecular marker will have a detection score of 100% if a peak in its XIC is aligned with XIC of all expected fragments according to the criteria above. To save computation time only three most abundant peaks in the XIC of every precursor are tested for concurrency with fragment peaks and then the XIC peak with the highest detection score is reported along with its score value and absolute retention time.

The correlative chromatogram peak intensity and the retention time-score values tuple for every marker ion are both active numbers. One click onto them displays the overlay of the precursor and fragment chromatograms for a rapid visual evaluation. An example of aligned chromatograms as displayed by the Arcadiate graphical interface is shown below.

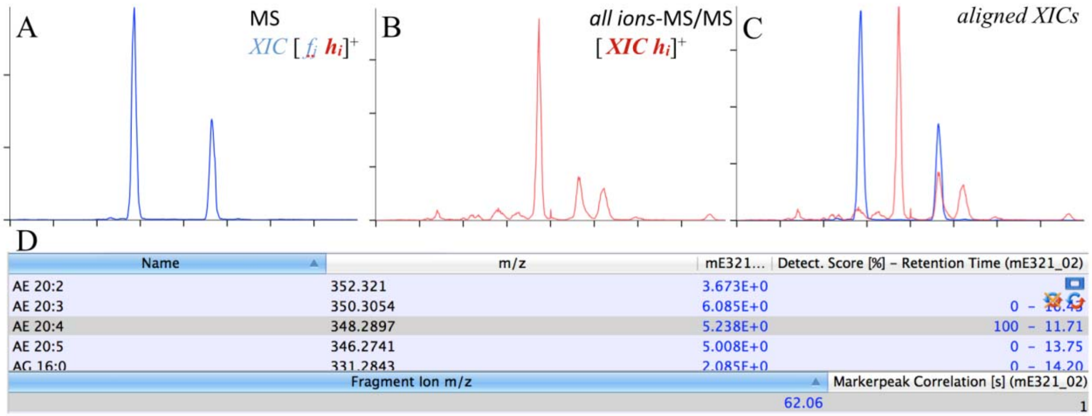

Here A, B and C are XICs of the precursor (molecular marker; in blue), fragment (in red) and their alignment, respectively. Interface panel D displays the compound name (here *N*-acetylethanolamine NAE 20:4); its *m/z* (348.2897 Th); the logarithmic abundance of correlative chromatogram of the precursor and its fragments; detection score (here 100%) and retention time (here 11.71 min) of the best correlating peak. The second table displays the fragment *m/z* (62.06 Th) and whether this fragment correlated with the precursor peak (1 for yes, 0 for no) for the precursor selected in the first column. Here mE321_02 is the name of the dataset used in this experiment.

Chemical synthesis of N-acylaspartate

All chemicals were obtained from commercial sources (Acros, Sigma,-Aldrich, Lancaster or Merck) and were used without further purification. Solvents for flash chromatography were obtained from VWR and

dry solvents were obtained from Sigma. Deuterated solvents were obtained from Deutero GmbH, Karlsruhe, Germany. All reactions were carried out using dry solvents under an inert atmosphere unless otherwise stated in the respective experimental procedure. TLC was performed on precoated plates of silica gel (Merck, 60 F254) using UV light (254 or 366 nm) or a solution of phosphomolybdic acid in EtOH (10 g phosphomolybdic acid, in 100 mL EtOH) for analysis. Preparative column chromatography was performed using silica form Merck, Darmstadt, Germany (silica 60, grain size 0.063-0.200 mm) with a pressure of 1 - 1.5 bar. ^{1}H- and ^{13}C-NMR-spectra were obtained on a 400 MHz Bruker UltraShield^{TM} spectrometer. Chemical shifts of ^{1}H- and ^{13}C-NMR-spectra are referenced indirectly to tetramethylsilane. *J* values are given in Hz and chemical shifts in ppm. Splitting patterns are designated as follows: s, singlet; d, doublet; t, triplet; q, quartett; m, multiplet; m_c, centered multiplet; b, broad. ^{13}C-NMR-spectra were broadband hydrogen decoupled. High resolution ESI mass spectra were recorded on a Q Exactive tandem mass spectrometer (Thermo Fisher Scientific, Bremen, Germany) in direct infusion mode using robotic ion source TriVersa Nanomate (Advion BioSciences, Ithaca NY).

Synthetic procedures and analytical data of synthesized compounds:

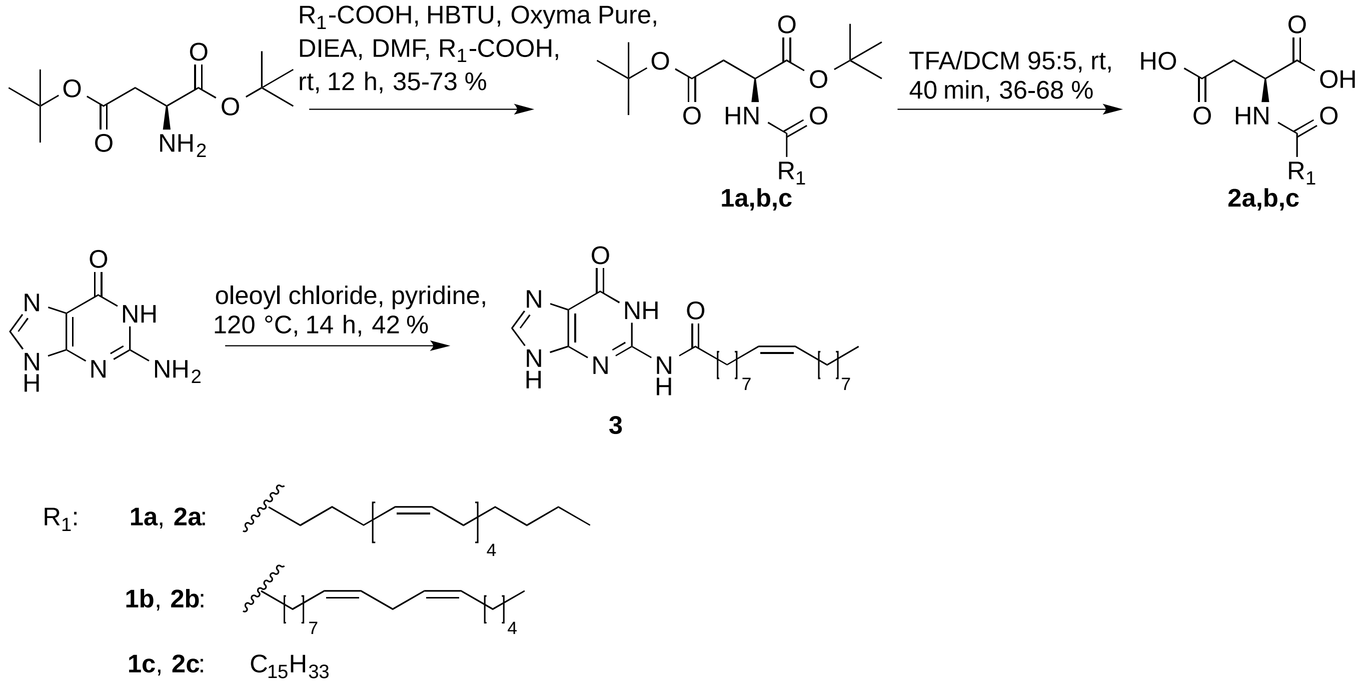

Synthesis of compounds **1a**,**b**,**c**; **2a**,**b**,**c** and **3**. **2a** is NAAsp 18:1, **2a** is NAAsp 18:2, **2a** is NAAsp 16:0.

General procedure for the synthesis of N-acyl-L-aspartic acid di(tert-butyl)ester derivatives 1a,b,c

A solution of ethyl cyano(hydroxyimino)acetate (oxyma pure) (0.15 eq) and HBTU (1.1 eq) in DMF (10 ml) was treated with a solution of the respective fatty acid (1.0 eq) in dry DMF (1 ml) while stirring at room temperature under an argon atmosphere. DIEA (2.0 eq) was added immediately afterwards and the reaction mixture stirred for 5 min. Stirring was continued for additional Over O/N after subsequent addition of L-aspartic acid di(tert-butyl)ester (1 eq). The reaction mixture was diluted with a mixture of EtOAc and H_2O (1:1, 100 ml) and the layers were separated. The organic layer was washed with H_2O mixed with 20 to 50 % brine (5 x 100 ml) and saturated NaCl solution (1 x 50 ml) and dried over Na_2SO_4. The solvent was removed under reduced pressure and the residue purified by flash chromatography using the eluent cyclohexane/EtOAc 3:1. The respective target compounds were obtained as colorless oils 1a, 1b or as white solid 1c.

N-ararchidonyl-L-aspartic acid di(*tert*-butyl)ester (**1a**)

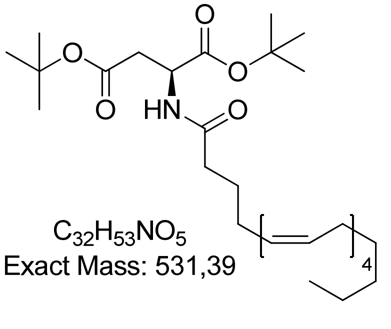

Yield: 510 mg, 59%.

^{1}H NMR (400 MHz, DMSO-D$_6$) δ = 8.14 (d, 1H, J = 9.0 Hz), 5.41-5.27 (m, 8H), 4.47 (m$_c$, 1H), 2.87-2.72 (m, 6H), 2.65 (dd, 1H, J = 16.2 Hz, 6.7 Hz), 2.54-2.46 (m, 3H), 2.12 (t, 2H, J = 7.4 Hz), 2.07-1.99 (m, 4H), 1.55 (m$_c$, 2H), 1.38 (s, 18H), 1.35-1.22 (m, 4H), 0.85 (t, 3H, J=6.5 Hz) ppm.

^{13}C NMR (101 MHz, DMSO-D$_6$) δ = 172.3, 170.4, 169.6, 130.4, 129.8, 128.6, 128.5, 128.5, 128.2, 128.1, 128.0, 81.1, 80.7, 49.7, 37.7, 35.0, 31.4, 29.2, 28.1, 28.0, 27.1, 26.8, 26.6, 25.8, 25.7, 25.6, 22.4, 14.4 ppm.

HR-MS (ESI positive) m/z found: 532.402, calculated for $C_{32}H_{54}NO_5^+$: 532.400.

N-linoyl-L-aspartic acid di(tert-butyl)ester (**1b**)

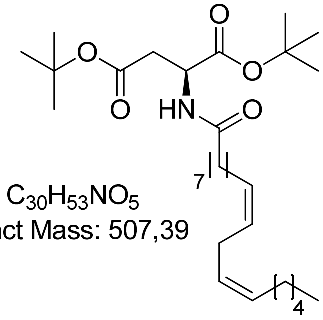

$C_{30}H_{53}NO_5$
Exact Mass: 507,39

Yield: 610 mg, 35 %.

^{1}H NMR (400 MHz, MeOD-D$_4$) δ = 5.45-5.29 (m, 4H), 4.63 (t, 1H, J = 6.1 Hz), 2.83-2.72 (m, 3 H), 2.66 (d, 1H, J = 16.1 Hz, 7.2 Hz), 2.24 (t, 2H, J = 7.1 Hz), 2.14-2.03 (m, 4H), 1.69-1.58 (m, 2H), 1.47 (s, 18 H), 1.44-1.28 (m, 14H), 0.93 (t, 3H, J = 6.2 Hz) ppm.

^{13}C NMR (101 MHz, MeOD-D$_4$) δ = 174.5, 170.0, 169.9, 129.6, 129.5, 127.7, 127.7, 81.6, 80.9, 49.7, 37.0, 35.4, 31.3, 29.4, 29.1, 29.0, 28.9, 28.9, 28.8, 27.0, 26.9, 26.8, 25.6, 25.2, 22.3, 13.1 ppm.

HR-MS (ESI positive) *m/z* found: 508.401, calculated for $C_{30}H_{54}NO_5^+$: 508.400199.

N-palmitoyl-L-aspartic acid di(tert-butyl)ester (**1c**)

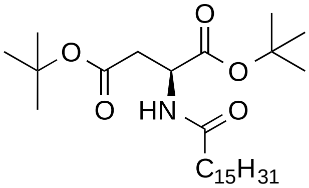

Chemical Formula: $C_{28}H_{53}NO_5$
Exact Mass: 483.39

Yield: 630 mg, 73 %

^{1}H NMR (400 MHz, MeOH-D$_4$) δ = 4.63 (dd, 1H, J = 7.1 Hz, 5.2 Hz), 2.77 (dd, 1H, J = 16.5 Hz, 5.2 Hz), 2.64 (dd, 1H, J = 16.5 Hz, 7.1 Hz), 2.24 (t, 2H, J = 7.0 Hz), 1.68-1.58 (m, 2H), 1.48 (s, 18H), 1.40-1.25 (m, 24H), 0.93 (t, 3H, J = 6.2 Hz) ppm.

^{13}C NMR (101 MHz, MeOH-D$_4$) δ = 174.6, 170.0, 169.9, 81.7, 81.0, 49.7, 36.9, 35.4, 31.7, 29.4, 29.4, 29.3, 29.1, 28.8, 26.9, 26.8, 25.6, 22.3, 13.0 ppm. Several signals in the aliphatic region overlap.

HR-MS (ESI positive*) m/z* found: 484.400, calculated for $C_{28}H_{54}NO_5^+$: 484.400.

NMR spectra of pure NAAsp showed extreme signal broadening in a wide temperature range, suggesting several conformations in equilibrium and were therefore excluded from the analytical data.

N-ararchidonyl-L-aspartic acid (**2a,** NAAsp 20:4)

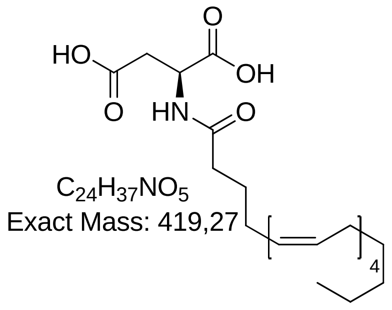

A solution of 168 mg (316 µmol) **1a** in TFA/DCM (10 ml, 95:5) was stirred for 40 min at room temperature. The reaction mixture was transferred onto a mixture of DCM and 0.5N HCl and the layers were separated. The organic layer was dried over Na_2SO_4 and the solvent removed under reduced pressure. The residue was purified by flash chromatography using the eluent $CHCl_3$/MeOH/H_2O 65:35:5 and the title compound obtained as a colorless solid (68 %, 90 mg).

HR-MS (ESI positive) *m/z* found: 420.2745, calculated for $C_{24}H_{38}NO_5^+$: 420.274449.

N-linoyl-L-aspartic acid (**2b** NAAsp 18:2)

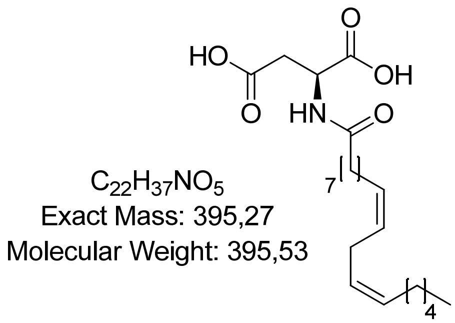

A solution of 170 mg (335 µmol) **1b** in TFA/DCM (10 ml, 95:5) was stirred for 40 min at room temperature. The reaction mixture was transferred onto a mixture of DCM and 0.5N HCl and the layers were separated. The organic layer was dried over Na_2SO_4 and the solvent removed under reduced pressure. The residue was purified by flash chromatography using the eluent $CHCl_3$/MeOH/H_2O 65:35:5 and the title compound obtained as a colorless solid (63 %, 83 mg).

HR-MS (ESI positive) *m/z* found: 396.2744, calculated for $C_{22}H_{38}NO_5$: 396.274449.

N-palmitoyl-L-aspartic acid (**2b** NAAsp 16:0)

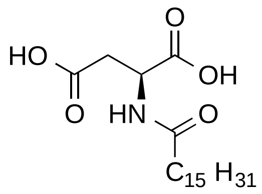

Chemical Formula: $C_{20}H_{37}NO_5$
Exact Mass: 371.27

A solution of 90 mg (186 µmol) **1c** in TFA/DCM (10 ml, 95:5) was stirred for 40 min at room temperature. The reaction mixture diluted wit DCM and the resulting precipitate washed several times with DCM (5 x 10 ml). The raw product was recrystallized from EtOH and the resulting solid washed with MeCN (3 x 2 ml). The title compound was obtained as a colorless solid (25 mg, 36 %)

HR-MS (ESI positive) *m/z* found: 372.2747, calculated for $C_{20}H_{38}NO_5$: 372.27449

Supplementary Table S1

Identification of N-acylethanolamines by multiple reaction monitoring (MRM) and parallel reaction monitoring (PRM) on the TSQ Vantage and Q Exactive mass spectrometers, respectively.

Analyte	Mass transitions		Collision energy[a]	S-lens[b]
	Precursor ion (*m/z*)	Product ion (*m/z*)	(eV)	(V)
N-acylethanolamine 12:0	244.2271	62.0604	14	104
N-acylethanolamine 14:0	272.2584	62.0604	14	107
N-acylethanolamine 16:0	300.2897	62.0604	16	111
N-acylethanolamine 16:1	298.274	62.0604	16	111
N-acylethanolamine 16:2	296.2584	62.0604	16	111
N-acylethanolamine 18:0	328.3210	62.0604	16	116
N-acylethanolamine 18:1	326.3053	62.0604	16	116
N-acylethanolamine 18:2	324.2897	62.0604	16	116
N-acylethanolamine 18:3	322.2740	62.0604	16	116
N-acylethanolamine 20:0	356.3523	62.0604	19	117
N-acylethanolamine 20:1	354.3366	62.0604	19	117
N-acylethanolamine 20:2	352.3210	62.0604	19	117
N-acylethanolamine 20:3	350.3053	62.0604	19	117
N-acylethanolamine 20:4	348.2897	62.0604	19	117
N-acylethanolamine 20:5	346.2740	62.0604	19	117
N-acylethanolamine 20:6	344.2584	62.0604	19	117
N-acylethanolamine 22:4	376.3210	62.0604	19	117
N-acylethanolamine 22:5	374.3053	62.0604	19	117
N-acylethanolamine 22:6	372.2897	62.0604	19	117

[a]For the analysis by MRM. For the analysis by PRM collision energy (nCE) was stepped at 22, 27 and 32 eV for all NAE species

[b]For the analysis by MRM. For PRM S-lens voltage was fixed at 50 V for all NAE species

Supplementary Table S2.

Endocannabinoid-related compounds identified in a rat kidney extract by AIF LC-MS/MS. New molecules for the first time identified in this work, are in red.

Compound[a]	Precursor ion $[M+H]^+$, *m/z*	Fragment ion $[M+H]^{+}$' *m/z*	Source organism and tissue[b]	Reference[c]
AG 16:0	331.2843	239.2368	RtKd	1
AG 16:1	329.2686	237.2212	RtKd	1
AG 16:2	327.2530	235.2055	RtKd	1
AG 18:0	359.3156	267.2681	RtKd	1
AG 18:1	357.2999	265.2525	RtKd/Dm	1, 2
AG 18:2	355.2843	263.2368	RtKd/Dm	1, 2
AG 18:3	353.2686	261.2212	RtKd	1
AG 20:3	381.2999	289.2525	RtKd	1
AG 20:4	379.2843	287.2368	RtKd/HsPl	1, 3
AG 22:4	407.3156	315.2681	RtKd	1
AG 22:6	403.2843	311.2368	RtKd	1
AG O-20:4	365.3050	273.2575	PcBr	4
NAAla 14:0	300.2533	90.0550	RtKd	1
NAAla 16:0	328.2846	90.0550	RtKd/Dm/RtBr	1, 2, 5
NAAla 18:0	356.3159	90.0550	RtKd/Dm/RtBr	1, 2, 5
NAAla 18:1	354.3003	90.0550	RtKd/Dm/RtBr	1, 2, 5
NAAla 18:2	352.2846	90.0550	RtKd/Dm/RtBr	1, 2, 5
NAAla 18:3	350.2690	90.0550	RtKd	1
NAAla 20:4	376.2846	90.0550	RtKd/RtBr/Bv	1, 5
NAAla 20:5	374.2690	90.0550	RtKd	1
NAArg 18:0	441.3799	175.1190	RtBr	5
NAAsn 16:0	371.2904	133.0608	RtKd	1
NAAsn 16:2	367.2591	133.0608	RtKd	1
NAAsn 18:0	399.3217	133.0608	RtKd	1
NAAsn 18:1	397.3061	133.0608	RtKd/RtBr	1, 5
NAAsn 18:2	395.2904	133.0608	RtKd	1
NAAsn 18:3	393.2748	133.0608	RtKd	1
NAAsn 20:3	421.3061	133.0608	RtKd	1
NAAsn 20:4	419.2904	133.0608	RtKd	1
NAAsn 20:5	417.2748	133.0608	RtKd	1
NAAsp 16:0	372.2744	134.0448	RtKd	1

NAAsp 18:2	396.2744	134.0448	RtKd	1
NAAsp 20:4	420.2744	134.0448	RtKd	1
NACys 18:1	386.2723	122.0270	RtKd	1
NADA 20:4	440.3159	137.0597	RtBr/Bv	5
NAE 12:0	244.2271	62.0600	RtKd	1
NAE 14:0	272.2584	62.0600	RtKd	1
NAE 16:0	300.2897	62.0600	RtKd/Dm/HsPl	1, 2, 3
NAE 16:1	298.2741	62.0600	RtKd	1
NAE 16:2	296.2584	62.0600	RtKd	1
NAE 18:0	328.3210	62.0600	RtKd/Dm/HsPl	1, 2, 3
NAE 18:1	326.3054	62.0600	RtKd/Dm/HsPl	1, 2, 3
NAE 18:2	324.2897	62.0600	RtKd/Dm/HsPl	1, 2, 3
NAE 18:3	322.2741	62.0600	RtKd	1
NAE 20:0	356.3523	62.0600	RtKd	1
NAE 20:1	354.3367	62.0600	RtKd	1
NAE 20:2	352.3210	62.0600	RtKd	1
NAE 20:3	350.3054	62.0600	RtKd	1
NAE 20:4	348.2897	62.0600	RtKd/HsPl	1, 3
NAE 20:5	346.2741	62.0600	RtKd	1
NAE 22:6	372.2897	62.0600	HsPl	3
NAGABA 16:0	342.3003	104.0706	RtBr	5
NAGABA 18:0	370.3316	104.0706	RtBr	5
NAGABA 18:1	368.3159	104.0706	Dm/RtBr	2, 5
NAGABA 18:2	366.3003	104.0706	Dm/RtBr	2, 5
NAGABA 20:4	390.3003	104.0706	RtBr/Bv	5
NAGABA 22:6	414.3003	104.0706	RtBr	5
NAGln 16:0	385.3061	147.0764	RtKd/RtBr	1, 5
NAGln 18:0	413.3374	147.0764	RtKd/RtBr	1, 5
NAGln 18:1	411.3217	147.0764	RtKd/RtBr	1, 5
NAGln 18:2	409.3061	147.0764	RtKd	1
NAGln 20:3	435.3217	147.0764	RtKd	1
NAGln 20:4	433.3061	147.0764	RtKd/RtBr	1, 5
NAGln 22:6	457.3061	147.0764	RtKd/RtBr	1, 5
NAGlu 16:0	386.2901	148.0604	RtKd/RtBr	1, 5
NAGlu 18:0	414.3214	148.0604	RtKd/RtBr	1, 5
NAGlu 18:1	412.3057	148.0604	RtKd/RtBr	1, 5
NAGlu 18:2	410.2901	148.0604	RtKd	1
NAGlu 18:3	408.2744	148.0604	RtKd	1
NAGlu 20:4	434.2901	148.0604	RtKd/RtBr	1, 5
NAGlu 22:6	458.2901	148.0604	RtBr	5
NAGly 12:0	258.2064	76.0393	RtKd	1

NAGly 14:0	286.2377	76.0393	RtKd	1
NAGly 16:0	314.2690	76.0393	RtKd/Dm/RtBr	1, 2, 5
NAGly 16:1	312.2533	76.0393	RtKd	1
NAGly 18:0	342.3003	76.0393	RtKd/Dm/RtBr	1, 2, 5
NAGly 18:1	340.2846	76.0393	RtKd/Dm/RtBr	1, 2, 5
NAGly 18:2	338.2690	76.0393	RtKd/Dm	1, 2
NAGly 18:3	336.2533	76.0393	RtKd	1
NAGly 20:4	362.2690	76.0393	RtKd/RtBr/Bv	1, 5
NAHis 16:0	394.3064	139.0502	RtKd/RtBr	1, 5
NAHis 18:1	420.3221	139.0502	RtKd/RtBr	1, 5
NAHis 18:2	418.3064	139.0502	RtKd	1
NAHis 20:4	442.3064	139.0502	RtKd/RtBr	1, 5
NAHis 20:5	440.2908	139.0502	RtKd	1
NAHis 22:6	466.3064	139.0502	RtKd/RtBr	1, 5
NALeu 16:0	370.3316	132.1019	RtKd/Dm/RtBr	1, 2, 5
NALeu 18:0	398.3629	132.1019	RtKd	1
NALeu 18:1	396.3472	132.1019	RtKd/Dm/RtBr	1, 2, 5
NALeu 18:2	394.3316	132.1019	RtKd/Dm	1, 2
NALeu 18:3	392.3159	132.1019	RtKd	1
NALeu 20:4	418.3316	132.1019	RtKd/RtBr	1, 5
NALeu 22:4	446.3629	132.1019	RtKd	1
NAMet 14:0	360.2567	150.0583	RtKd	1
NAMet 16:0	388.2880	150.0583	RtKd/Dm/RtBr	1, 2, 5
NAMet 16:1	386.2723	150.0583	RtKd	1
NAMet 18:0	416.3193	150.0583	RtBr/RtKd	1, 5
NAMet 18:1	414.3036	150.0583	RtKd/Dm/RtBr	1, 2, 5
NAMet 18:2	412.2880	150.0583	RtKd/Dm	1, 2
NAMet 18:3	410.2723	150.0583	RtKd	1
NAMet 20:0	444.3506	150.0583	RtKd	1
NAMet 20:4	436.2880	150.0583	RtKd	1
NAMet 20:5	434.2723	150.0583	RtKd	1
NAMet 22:4	464.3193	150.0583	RtKd	1
NAMet 22:5	462.3036	150.0583	RtKd	1
NAPhe 16:0	404.3159	166.0863	RtKd/Dm/RtBr	1, 2, 5
NAPhe 18:0	432.3472	166.0863	RtKd	1
NAPhe 18:1	430.3316	166.0863	RtKd/Dm/RtBr	1, 2, 5
NAPhe 18:2	428.3159	166.0863	RtKd/Dm	1, 2
NAPhe 20:4	452.3159	166.0863	RtKd	1
NAPhe 20:5	450.3003	166.0863	RtKd	1
NAPhe 22:6	476.3159	166.0863	RtBr	5
NAPro 16:0	354.3003	116.0706	RtBr	5

NAPro 18:0	380.3159	116.0706	RtBr	5
NAPro 18:1	382.3316	116.0706	RtBr	5
NASer 16:0	344.2795	106.0499	RtKd/Dm/RtBr	1, 2, 5
NASer 18:0	372.3108	106.0499	RtKd/Dm/RtBr	1, 2, 5
NASer 18:1	370.2952	106.0499	RtKd/Dm/RtBr	1, 2, 5
NASer 18:2	368.2795	106.0499	RtKd/Dm/RtBr	1, 2, 5
NASer 18:3	366.2639	106.0499	RtKd	1
NASer 20:4	392.2795	106.0499	RtBr/RtKd	1, 5
NASer 24:6	444.3108	106.0499	RtKd	1
NATau 16:0	364.2516	126.0219	RtBr	5
NATau 18:0	392.2829	126.0219	RtBr	5
NATau 18:1	390.2673	126.0219	RtBr	5
NATau 18:2	388.2516	126.0219	RtBr	5
NATau 20:4	412.2516	126.0219	RtBr	5
NATau 22:6	436.2516	126.0219	RtBr	5
NAThr 16:0	358.2952	120.0655	RtBr/RtKd	1, 5
NAThr 18:0	386.3265	120.0655	RtKd	1
NAThr 18:1	384.3108	120.0655	RtBr/RtKd	1, 5
NAThr 18:2	382.2952	120.0655	RtKd	1
NAThr 20:2	410.3265	120.0655	RtKd	1
NAThr 20:4	406.2952	120.0655	RtKd	1
NATrp 14:0	415.2955	205.0972	RtKd	1
NATrp 16:0	443.3268	205.0972	RtKd/Dm/RtBr	1, 2, 5
NATrp 18:0	471.3581	205.0972	RtKd/Dm/RtBr	1, 2, 5
NATrp 18:1	469.3425	205.0972	RtKd/Dm/RtBr	1, 2, 5
NATrp 18:2	467.3268	205.0972	RtKd/Dm	1, 2, 5
NATrp 18:3	465.3112	205.0972	RtKd	1
NATrp 20:4	491.3268	205.0972	RtKd	1
NATyr 16:0	420.3108	182.0812	Dm/RtBr	2, 5
NATyr 18:0	448.3421	182.0812	Dm/RtBr	2, 5
NATyr 18:1	446.3265	182.0812	Dm/RtBr	2, 5
NATyr 18:2	444.3108	182.0812	Dm	2
NATyr 20:4	468.3108	182.0812	RtBr	5
NATyr 24:6	520.3421	182.0812	RtKd	1
NAVal 12:0	300.2533	118.0863	RtKd	1
NAVal 14:1	326.2690	118.0863	RtKd	1
NAVal 16:0	356.3159	118.0863	RtKd/Dm/RtBr	1, 2, 5
NAVal 16:1	354.3003	118.0863	RtKd	1
NAVal 16:2	352.2846	118.0863	RtKd	1
NAVal 18:0	384.3472	118.0863	RtKd/Dm/RtBr	1, 2, 5
NAVal 18:1	382.3316	118.0863	RtKd/Dm	1, 2

NAVal 18:2	380.3159	118.0863	RtKd/Dm/RtBr	1, 2, 5
NAVal 20:3	406.3316	118.0863	RtKd	1
NAVal 22:4	432.3472	118.0863	RtKd	1
NAVal 22:6	428.3159	118.0863	RtKd	1

[a]Annotation of ERC classes.

[b]Abbreviations:
Source organisms: Rt; rat; Dm; Drosophila; Hs; human, Bv; bovine; Pc, porcine.
Source tissues and biofluids: Br; brain, Pl; blood plasma; Kd; kidney,

[c] References for Supplementary Table S2

1: this work

2: Tortoriello G, Rhodes BP, Takacs SM, Stuart JM, Basnet A, Raboune S, Widlanski TS, Doherty P, Harkany T, Bradshaw HB (2013) Targeted lipidomics in Drosophila melanogaster identifies novel 2-monoacylglycerols and N-acyl amides. PloS One 8: e67865

3: Sipe JC, Scott TM, Murray S, Harismendy O, Simon GM, Cravatt BF, Waalen J (2010) Biomarkers of endocannabinoid system activation in severe obesity. PLoS One 5: e8792

4: Hanus L, Abu-Lafi S, Fride E, Breuer A, Vogel Z, Shalev DE, Kustanovich I, Mechoulam R (2001) 2-arachidonyl glyceryl ether, an endogenous agonist of the cannabinoid CB1 receptor. Proc Natl Acad Sci U S A 98: 3662-3665

5: Tan B, O'Dell DK, Yu YW, Monn MF, Hughes HV, Burstein S, Walker JM (2010) Identification of endogenous acyl amino acids based on a targeted lipidomics approach. J Lipid Res 51: 112-119

References for Supplementary Materials

Gattinoni S, De Simone C, Dallavalle S, Fezza F, Nannei R, Amadio D, Minetti P, Quattrociocchi G, Caprioli A, Borsini F, Cabri W, Penco S, Merlini L, Maccarrone M. (2010) Enol carbamates as inhibitors of fatty acid amide hydrolase (FAAH) endowed with high selectivity for FAAH over the other targets of the endocannabinoid system. ChemMedChem. 5:357-360

Huang SM, Bisogno T, Trevisani M, Al-Hayani A, De Petrocellis L, Fezza F, Tognetto M, Petros TJ, Krey JF, Chu CJ, Miller JD, Davies SN, Geppetti P, Walker JM, Di Marzo V (2002) An endogenous capsaicin-like substance with high potency at recombinant and native vanilloid VR1 receptors. Proc Natl Acad Sci USA 99: 8400-8405

Pucci M, Pasquariello N, Battista N, Di Tommaso M, Rapino C, Fezza F, Zuccolo M, Jourdain R, Finazzi Agrò A, Breton L, Maccarrone M (2012) Endocannabinoids stimulate human melanogenesis via type-1 cannabinoid receptor. J Biol Chem. 287: 15466-15478

Tan B, Yu YW, Monn MF, Hughes HV, O'Dell DK, Walker JM (2009) Targeted lipidomics approach for endogenous N-acyl amino acids in rat brain tissue. J Chromatogr B Analyt Technol Biomed Life Sci 877: 2890-2894

Supplementary Table S3

Quantification of major classes and individual molecules of endocannabinoid related compounds in the rat kidney extract

ECR compounds	Content in rat kidney, fmol/mg[a]
1-AG 16:0	103.3
1-AG 18:2	493.8
1-AG 20:4	59.2
Total 1-AG:	656.3
2-AG 16:0	99.0
2-AG 18:2	1815.1
2-AG 20:4	277.3
Total 2-AG:	2191.4
NAE 16:0	28.7
NAE 18:2	23.7
NAE 20:4	5.1
Total NAE:	57.5
NAGly 16:0	280.3
NAGly 18:2	193.8
NAGly 20:4	61.8
Total NAGly:	535.9
NAAsp 16:0	14.8
NAAsp 18:2	5.5
NAAsp 20:4	2.4
Total NAAsp:	22.7

[a]femtomoles of ERC species per mg of rat kidney tissue. RSD was below 10% (*n*=2)
